# Iron intake among Lebanese women: sociodemographic factors, iron-rich dietary patterns, and preparation of hummus, a Mediterranean dish

**DOI:** 10.29219/fnr.v65.5556

**Published:** 2021-01-06

**Authors:** Nour Doumani, Jacqueline Maalouly, Elias Bou-Maroun, Nicolas Sok, Philippe Cayot, Maya Tueni

**Affiliations:** 1 Department of Biology, Nutrition & Dietetics, Faculty of Sciences II, Lebanese University, Fanar, Lebanon; 2 Department of Chemistry & Biochemistry, Faculty of Sciences II, Lebanese University, Fanar, Lebanon; 3 UMR PAM Food and Microbiological Processes, University of Burgundy Franche Comté/AgroSup Dijon, Dijon, France

**Keywords:** micronutrient intake deficiency, plant-based food, nonheme iron, Mediterranean and middle eastern countries, iron bioavailability

## Abstract

**Background:**

Plant-based foods such as hummus are alternative to animal protein, and when properly prepared, they help to alleviate nutritional iron deficiency that leads to anemia, a global health problem.

**Objective:**

The objective was to assess iron intake among Lebanese women and related participant’s characteristics, discern iron-rich dietary patterns, evaluate their association with nutrients intake and participant’s sociodemographic characteristics, and identify the women preparing hummus traditionally and properly for an enhanced iron bioavailability.

**Design:**

A cross-sectional study of 400 Lebanese women (18–74 years old) was conducted in Lebanon. Data from a questionnaire, including sociodemographic and health characteristics, dietary intake, and hummus preparation and consumption, were collected. Dietary data were obtained by a food frequency questionnaire and a 24-h recall. Dietary patterns were identified by principal component analysis. Linear regression and binomial logistic regression models were used to explore the association between the intake of dietary iron, its patterns, and the participants’ characteristics.

**Results:**

About 60% of the women had iron intake deficiency, especially with lower income (odds ratio [OR] = 1.88, 95% confidence interval [CI]: 1.107, 3.194). Four iron-rich dietary patterns were identified: legumes; organ/lunch meat and chicken; canned fish; and beef and hummus. The factor scores of the latter were positively correlated with protein, vitamin C, iron, folate, vitamin B12, and vitamin A with *r* = 0.195 and *P* < 0.01 for all. No significant difference was shown among the women’s sociodemographic characteristics for the consumption of the hummus-related pattern. Only 9.2 and 22.7% of the women considered proper preparation of chickpea and hummus, respectively, which significantly (*P* < 0.05) correlated with older women (66.7%).

**Discussion & Conclusion:**

The majority of the Lebanese women still have iron intake deficiency and the minority reported proper preparation of hummus. Intervention programs spreading awareness among Lebanese women are needed for encouraging adequate iron intake and considering proper steps to improve iron bioavailability from plant-based food.

## Popular scientific summary

- Iron intake is still inadequate to dietary recommendations among Lebanese women from a nationally representative sample by age and region.
- Women with lower income are significantly more prone to have iron intake deficiency.
- Nonheme iron inhibitors are frequently consumed around mealtimes.
- Dietary pattern including hummus is related to higher intake of protein, vitamin C, and anemia-related nutrients.
- Less than the quarter of the women consider a proper preparation of hummus that enhances iron bioavailability.

Iron deficiency, accounting for 25% of anemia causes, is the most common nutritional disorder affecting people in developed as well as developing countries (1). This health condition is defined by an inadequate amount of iron and results in anemia. Women of reproductive age are at high risk of developing iron-deficiency anemia, especially in developing countries (2). In Lebanon, 16% of the childbearing-aged women have anemia and 27.2% have iron deficiency according to Al Khatib et al. (2006) (3); one major reason for developing them is attributed to the inadequate dietary intake of iron (4).

Two dietary sources of iron exist: animal and plant sources (5). Animal products such as meat poultry and fish contain heme iron, the form of iron easily absorbed by the human body. Although the consumption of animal-based products is important to provide the better form of dietary iron for absorption (heme iron), the climate researchers recommend a decrease of animal consumption since farming and livestock production are threatening to the environment and health (6–8). On the contrary, plant products such as legumes and pulses are eco-friendly, good for health (9–11), and have a high protein content. Legumes and pulses are good sources of iron. However, they contain the nonheme iron, which is the harder form of iron to be absorbed (12).

One famous traditional Lebanese dish based on legumes and consumed internationally is ‘hummus’, a spread made from mashed boiled chickpeas and blended with tahini, lemon juice, and garlic (13, 14). Hummus is not only an alternative to meat but also a dip that provides a good source of iron (2.4 mg of iron per 100 g hummus) (15). As the other plant-based products, the iron contained in hummus is poorly absorbed (nonheme iron). Nonheme iron assimilation is influenced by several factors such as the iron status and the gastric pH of the consumer, as well as dietary factors (16). The dietary factors include enhancers and inhibitors of iron bioavailability. On the one hand, some of these factors are endogenous to the food itself (17) and their content could be influenced through the processing and formulation of the same food (18–20). Regarding iron bioaccessibility from chickpea, soaking was shown to reduce antinutrients from chickpea (21–23). Cooking chickpea by a pressure cook was shown to be more effective in reducing the content of inhibitors than boiling (18, 24, 25). One study showed that the decortication of chickpea reduced antinutrients and improved the bioavailability of iron (26). Regarding iron bioavailability from hummus, a recent study by Doumani and coworker (27) has shown that among all the steps for hummus preparation (soaking, bicarbonate addition, cooking, decortication of chickpea, lemon juice tahini, and garlic addition), only pressure cooking of chickpea, lemon juice, and tahini had a significant influence on its iron bioavailability. While pressure cooking and lemon juice addition were positively correlated with iron bioavailability, tahini addition was negatively correlated (27). On the other hand, some factors are exogenous to the food as they come from other items consumed concomitantly within the same meal (28, 29) and their interaction could be improved by making appropriate food combinations such as eating iron-rich food with vitamin-C-rich juices and avoiding polyphenol-rich food such as coffee. Therefore, food preparation (processing and formulation) and combination are important to consider as they influence iron bioavailability from plant-based food such as chickpea and hummus.

To the best of our knowledge, there is no study that investigated if the women are aware of proper preparation of plant-based food, particularly chickpea and hummus. Thus, this will be investigated in this study by conducting a survey on a sample of Lebanese women across all the Lebanese regions. Any sociodemographic characteristics association will be regarded as well. Our study targets the women as they are at high risk to develop iron deficiency and are still considered the prime source of cooking knowledge in Lebanese households and other countries too (30–32). The present study is divided into two sections. In the first section, the objectives are to 1) investigate iron intake among the Lebanese women and its relation with participants’ characteristics and 2) distinguish the iron-rich dietary patterns and asses their association with nutrients intake and participant’s sociodemographic characteristics. In the second section, the objective is to identify women who prepare hummus traditionally and investigate if women take into consideration the proper preparation of chickpea and hummus for an enhanced iron bioavailability.

## Materials and methods

### Study design and sample

A cross-sectional survey was carried out between May 2018 and October 2018, on a representative subpopulation of 400 women based on age and regions according to the *Central Administration of Statistics* of Lebanon in 2009 (33). The survey was a three-stage stratified, cluster sample that covered each of Lebanon’s six administrative divisions (called governorates). The interviewees aged 18–74 years were randomly selected from the six governorates in Lebanon: Beirut, Mount Lebanon, North, Beqaa, South, and Nabatiyeh. The sample size for this study was calculated using the following formula: $n=\frac{N}{1+N{\left(e\right)}^{2}}$, where *n* is the sample size, *N* (1.9 million Lebanese women) is the population size, and *e* is the level of precision at 5% (34). Women were interviewed randomly in each region. From each household, one woman, young, middle aged, or old adult, was invited to participate in the survey. The exclusion criteria included pregnant or lactating women, and women with cognitive impairments and emotional problems. The participants were informed about the objectives of the survey and about their right to withdraw from the study at any time; 2% of the women dropped out from the study for a lack of time. The results were anonym and confidential. The study did not expose the participants to any risk.

### Data collection procedures

The questionnaire was pretested on 30 women and then refined to further improve its validity and reliability. The questions were asked in Arabic-Lebanese (local language). Trained dietitians conducted face-to-face interviews and entered the data immediately through a computerized administration mode on their tablets. Each interview took an average of 20 min. The questionnaire was divided into three parts: 1) sociodemographic and health characteristics, 2) dietary intake, and 3) hummus preparation and combination with food and beverage items for consumption.

### Sociodemographic characteristics

The questionnaire covered information on socio-demographic characteristics, and it included six close-ended questions: age (young: 18–39 years, middle aged: 40–59 years, old adult: 60–74 years), residence area (rural or urban), marital status (married or not), university level (yes or no), work status (housewife or other, including working women and students), monthly family income (≤500$ or >500$). Other characteristics such as body mass index (BMI), physical activity, and diet (normal or vegetarian) were covered as well. For the daily intake assessment part, women were classified into premenopausal (18–49 years) and menopausal women (50 years and more) respecting the recommended dietary requirements for each group. Weight and height were collected to calculate BMI as follows: $\text{BMI}=\frac{\text{weight}\;(\text{kg})}{\text{height}\;({\text{m}}^{2})}$ (35). Concerning the physical activity, women were classified as sedentary or not active (doing no extra activity to independent living), or active including moderately active (doing extra activity equivalent to 2.5–5 km walking/day) and rigorously active (doing extra activity equivalent to more than 5 km walking/day) (36).

### Dietary intake

The frequency of the consumption of iron-rich food and modulators of iron bioavailability was assessed by a qualitative food frequency questionnaire (FFQ) on monthly, weekly, or daily basis during the past 6 months with a total of 24 close-ended questions. The questionnaire included 17 food sources of iron divided into two categories according to the form of iron they contain. The first group included foods providing the heme and nonheme iron; these are exclusively from animal sources and included beef, organ meet, lunch meat, chicken, tuna, and sardines. The second group included foods providing solely nonheme iron; these originate from plants (except for the eggs) and covered beans, lentils, and chickpea presented as total legumes, hummus, green leafy vegetables, seeds, nuts, dried fruit, and carob molasses. The questionnaire also included seven modulators of iron bioavailability adapted from models in other Lebanese studies (37, 38). Iron enhancers included vitamin-C-rich fruit and juices (lemon and orange juices), whereas iron inhibitors included calcium, polyphenols, tannins, phytate, and/or fiber-rich food (milk, yogurt, coffee/tea, soft drinks, and whole wheat bread). The questionnaire was validated based on the ‘Food-Based Dietary Guideline Manual for Promoting Healthy Eating in the Lebanese Adult Population: Lebanon’ (37). As for the assessment of the daily intake of calories, iron, and nutrients influencing iron bioavailability (ascorbic acid, vitamin A, fibers, and calcium), a single 24-h dietary recall was employed. The 24-h dietary recall is an assessment tool of the type and quantity of food and beverage consumed in the past 24 h per a typical day of the week. The information obtained was analyzed by a nutritional software package ‘Nutrilog 2018’ (A French Nutrition Software Company) (38), and the nutrient intakes were calculated based on two food composition databases: The Middle Eastern food composition table for local dishes (39) and the United States Department of Agriculture (USDA) food composition table for international dishes (40).

### Distinction of iron-rich dietary patterns

The principal component analysis (PCA) was used to identify iron-rich dietary patterns using the frequency dietary intake of 17 iron-rich food items. PCA is a data-driven method that reveals foods that are frequently consumed together by pooling food items on the basis of the degree to which they are correlated with one another (41). In this study, the PCA revealed the food items found to be frequently consumed together identified by the food frequency assessment. Before running the PCA procedure, the correlation matrix among the 17 iron-rich food groups was visually and statistically examined to justify undertaking the analysis. The Kaiser–Meyer–Olkin measure of sampling adequacy test had a score of 0.656 (>0.6), and the *χ* ^2^ for the Bartlett test of sphericity was significant as the *P*-value was less than 0.05, indicating that the correlation among the variables was sufficiently strong for a PCA. The number of components (factors) was retained based on three criteria: 1) the Kaiser criterion (eigenvalues >1); 2) inflection point of the scree plot; and 3) interpretability of factors (42). To achieve a simple structure with greater interpretability, the factors were rotated using an orthogonal transformation known as Varimax rotation (41). Factor loadings indicated the strength and direction of the relationship between the food items and each dietary pattern. These were labeled on the basis of the iron-rich food items having a rotated factor loading >0.5. The multiple regression approach was used to calculate the factor scores. Each participant received a factor score for each iron-rich dietary pattern, showing the degree to which each individual’s iron food consumption corresponded to the identified pattern.

### Hummus preparation and consumption

The 12 questions related to hummus preparation and consumption with food and beverage included hummus preparation traditionally (from raw ingredients), not traditionally (from industrially cooked chickpeas), or neither of these (do not prepare hummus). For the traditional preparation, further questions were addressed, including precooking of chickpeas (soaking, with bicarbonate, discarding the water), cooking (boiling or pressure cooking), and post-cooking (decortication of chickpea). For all the women who reported to prepare hummus, they were asked about the proportions of ingredients used for making hummus (tahini, garlic, and lemon juice). All the women were asked about their preferences for food and beverages items combined with hummus consumption (meat or vegetables, refined or unrefined bread, and soft drinks or yogurt or vitamin-C-rich juices).

### Proper chickpea and hummus preparation

To evaluate if women properly prepare chickpea and hummus, we have considered the processing and formulation steps that have been proved in the literature (18, 24, 25, 27) to improve iron bioaccessibility and bioavailability from chickpea and hummus, respectively. Regarding chickpea, three steps were considered: soaking chickpea, cooking chickpea with a pressure cook, and decortication of chickpea. Regarding hummus, three steps were considered: pressure cooking, more lemon juice addition, and less tahini.

### Statistical analysis

Descriptive statistics were calculated and results are expressed as mean values ± standard deviations (SD) for continuous variables and percentages for categorical variables. As the energy-adjusted nutrient intakes were not normally distributed, they were presented as quartiles. To study the relationship between categorical variables, the *χ* ^2^-test was used. Dietary consumption of macro- and micronutrients was analyzed using professional nutritional software NutriLog 3.01 (38). A multiple linear regression was used to assess the association between the normalized total iron intake (dependent continuous variable) and the participants’ sociodemographic characteristics (independent categorical variables), as well as between the normalized iron intake adequacy percentage (dependent continuous variable) and the participants’ sociodemographic characteristics (independent categorical variables). A binomial logistic regression analysis was conducted in order to assess the role of the participants’ sociodemographic characteristics (predictors) on the iron intake deficiency (dichotomous outcome). The odds ratios (ORs) and their 95% confidence intervals (CIs) for each of the variables were generated. To study the association between iron-rich dietary patterns and the nutrient intake, Spearman’s rank correlation coefficients with two-sided significance levels were calculated between the factor scores of each pattern and the energy-adjusted nutrient intakes. This nonparametric test was used as both continuous variables (factor scores and energy-adjusted nutrient intakes) were not normally distributed. To study the association between the factor scores of the iron-rich dietary patterns (continuous, dependent variables) and the sociodemographic characteristics (dichotomous, independent variables), a multiple linear regression analysis was applied to assess their associations. For all the linear regressions, the assumptions were fulfilled to assess the validity of the final regression models including the residual analysis and collinearity diagnosis. The Statistical Package for the Social Sciences version 21.0 (SPSS for Windows; SPSS Inc., Chicago, IL, USA) was used. A *P*-value less than 0.05 was considered significant.

## Results

### Descriptive characteristics of the sample

The socio-demographic and health characteristics of the sample are described in Table 1. Out of the 400 study participants, slightly more than the half were young adults (56%), from urban areas (54%), hold a higher academic degree (52%), and with busy lifestyles (65%). Concerning health characteristics of the women, the overall means for the weight and height were 66.79 ± 11.3 kg and 1.64 ± 0.003 m, respectively. The average BMI was 24.95 ± 0.21 kg/m^2^, and about half the women had a BMI of <25 kg/m^2^ (53.25%) with almost all of them (93%) having a normal BMI. Furthermore, slightly more than half the women had a moderate or vigorous activity (54.75%) and almost all of them followed a normal diet.

**Table 1:** Descriptive socio-demographic and health characteristics of the studied sample (*N* = 400), Lebanon 2018

Characteristic	Frequency	Percentage	Characteristic	Frequency	Percentage
	*n*	%		*n*	%
**Age group^a^**			**Monthly total income**		
Young adult	224	56.0	≤500$	120	30.0
Middle-aged/old adults	176	44.0	>500$	280	70.0
**Residence area**			**BMI^c^**		
Rural	184	45.9	<25 kg/m^2^	213	53.25
Urban	216	54.1	≥25 kg/m^2^	187	46.75
**Marital status**			**Physical activity^d^**		
Never married	168	42.0	Not active	181	45.25
Married	232	58.0	Active	219	54.75
**University level**			**Diet**		
No	190	47.5	Vegetarian	7	1.8
Yes	210	52.5	Normal	393	98.2
**Occupational status^b^**					
Housewife	141	35.2			
Other	259	64.8			

a Young adult: aged 18–39 years old; middle aged: 39–59 years old; old adults: 60–74 years old.

b Occupational status: other means working women and students.

c BMI is the body mass index and is obtained by dividing the weight (kg) by height square (m). It is divided into two categories: underweight (<18.5 kg/m^2^) and normal (18.5–24.9 kg/m^2^) which are <25 kg/m^2^ and another category which are >25 kg/m^2^ including overweight (25–29.9 kg/m^2^) and obese (≥30 kg/m^2^) (35).

d Physical activity: not active includes the sedentary women with no extra activity, and active includes the women who do an activity (moderate or intense) equivalent to at least 2–5 km walking/day (36).

### Iron intake data

Consumption frequency of iron rich food based on FFQ The percentages of the participants consuming iron-rich products are shown in Table 2. Our study shows that beef and chicken are the primary sources of heme iron intake as more than 90% of the women reported their consumption frequently (at least once a week). Concerning the nonheme iron intake, hummus like legumes and green leafy vegetables constitutes a major source of the nonheme iron as at least 80% of the women reported its frequent consumption (at least once a week). Frequency of heme iron intake in our panel is considerable (57.3% with *P* = 0.038), but it is less important than the frequency of the nonheme iron intake (65.9% with *P* = 0.038). Thus, the consumption of nonheme iron bioavailability modulators around mealtimes is investigated.

**Table 2:** Frequency of consumption of iron-rich food, iron enhancers, and iron inhibitors reported by the total study population of the Lebanese women aged 18–74 years (*N* = 400) and expressed in % of consumers, Lebanon 2018

Food item		% of consumers Frequency of consumption				
		Monthly	Weekly 1–2 … 3–6		Daily	*P*
**Iron-rich food in both forms**	Iron content (mg)/serving)^a^					0.038*
**Heme and nonheme iron^b^**	40% heme (H) vs. 60% nonheme (NH)	42.6	26.0	30.1	1.2	
Beef, tenderloin, and cooked (90 g)	3	9.2	23.5	64.6	2.7	
Organ meat (90 g)	5.2–9.9	76.0	20.2	3.2	0.6	
Lunch meat (100 g)	1.9	43.8	28.5	25.4	2.3	
Chicken (100 g)	1.1	4.8	21.8	72.2	1.2	
Tuna (1 can)	2.5	44.8	43.0	12.0	0.2	
Sardines (1 can)	2.5	77.2	19.2	3.4	0.2	
**Only nonheme iron^c^**	100% NH	34.1	33.6	24.5	7.8	
Egg (1 large of 50 g)	0.6	24.5	47.2	25.8	2.5	
Breakfast cereal fortified (^3^⁄_4_ cup)	4.5	63.0	16.5	17.8	2.7	
Total legumes cooked (1 cup)^d^	5.5	15.7	32.3	47.5	4.5	
Hummus (100 g)	2.4	19.2	58.8	22	0.0	
Green leafy vegetables (1 cup)^e^	0.4	12.4	21.8	28	37.8	
Dried fruit (^1^⁄_4_ cup)^f^	0.8	57.5	25.0	13.5	4.0	
Roasted pumpkin seeds (^1^⁄_4_ cup)	1.4–4.7	47.0	28.8	18.7	5.5	
Nuts (^1^⁄_4_ cup)	1.3–2.2	33.8	38.5	22.3	5.4	
Carob molasses (1 tablespoon)	3.5	84.2	11.0	3.7	1.1	
**Iron-enhancing or iron-inhibiting food around mealtimes**						0.010*
**Iron enhancers^g,h^**		52.3	42.9		4.8	
Lemon/orange juice	–	55.2	40.0		4.2	
Citrus fruit		48.8	45.7		5.5	
**Iron inhibitors^g,i^**		36.9	46.1		17.0	
Milk	–	55.2	37.6		7.2	
Yogurt	–	21.0	71		8.0	
Coffee or tea	–	23.7	36.3		40.0	
Soft drinks with caffeine	–	40.2	49.6		10.2	
Whole wheat bread	–	44.5	36.0		19.5	

a References: (14, 15, 38).

b Animal sources, except eggs, are assumed to contain 40% heme iron and 60% nonheme iron (43).

c Plant sources, in addition to eggs, contain 100% nonheme iron.

d Iron content in legumes is the mean iron content from chickpea, lentils, and beans (commonly consumed legumes).

e Iron content in green leafy vegetables is the mean iron content from raw: arugula, chicory, chard, kale, romaine lettuce, and spinach (commonly consumed leafy vegetables).

f Iron content in dried fruits is the mean iron content from dates, dried apricots, and dried raisins (commonly consumed dried fruits).

g Iron absorption modulators consumed around meal times (i.e. breakfast, lunch, and dinner).

h The percentage of women consuming iron enhancers is the mean percentage of the women consuming lemon/orange juice and citrus fruit.

i The percentage of women consuming iron inhibitors is the mean percentage of the women consuming milk, yogurt, coffee/tea, soft drinks, and whole wheat bread.

* Significance level set at *p*-value < 0.05

#### Consumption frequency of dietary modulators of nonheme iron bioavailability based on FFQ

The percentages of the participants consuming iron-enhancing and iron-inhibiting food items around mealtimes are shown in Table 2. There is a significantly higher percentage of the participants consuming frequently iron inhibitors around mealtimes than the percentage of participants consuming frequently iron enhancers (63.1% vs. 47.7% with *P* = 0.010). This means that nonheme iron-rich food generally consumed during the meals must be more prone to interact with inhibitors of iron bioavailability (*e.g.,* polyphenols, tannins, and calcium) instead of enhancers (*e.g.,* vitamin C).

#### Dietary intake for iron and other anemia-related nutrients based on 24-h recall

The mean intake, mean intake adequacy, and percentage of women with dietary intake deficiency for energy-adjusted nutrients are shown in Table 3. The mean intake adequacy percentage for iron is considerably lower among women of childbearing age (65.58% of recommended dietary allowance [RDA]) than postmenopausal women (142.77% of RDA) but is almost similar for other anemia-related nutrients: folate (59.6% of RDA), vitamin B12 (125.9% of RDA), and vitamin A (56% of RDA), as well as ascorbic acid (82.6% of RDA). About 60% of the childbearing-aged women have iron intake deficiency, and more than 70% of them have intake deficiencies for folate (70.6%) and vitamin A (81.6%).

**Table 3:** Recommended dietary allowance (RDA), daily intake as quartiles and mean values, mean intake adequacy, and percentage of women with dietary intake deficiency for selected nutrients (*N* = 400), Lebanon 2018

Nutrient^a^	Nutrient intake represented in quartiles and mean values					Mean intake adequacy	% of women with intake deficiency^e^
	RDA^b^	Q1	Q2	Q3	Mean ± standard deviation (SD)	% ± SD	
Total iron (mg)							
	18^c^	9.67	11.33	13.51	11.80 ± 3.56	65.58 ± 19.77	58.50
	8^d^	9.44	11.22	13.07	11.42 ± 2.45	142.77 ± 30.64	1.00
Ascorbic acid (mg)							
	75^c^	54.46	60.85	69.40	62.72 ± 14.38	83.63 ± 19.18	10.70
	75^d^	53.37	60.24	67.51	61.18 ± 9.62	81.58 ± 12.82	9.00
Folate (µg)							
	400^c^	196.68	232.79	279.55	242.51 ± 75.65	60.63 ± 18.91	70.60
	400^d^	191.82	230.25	270.20	234.33 ± 53.02	58.58 ± 13.25	73.00
Vitamin B12 (µg)							
	2.4^c^	2.20	2.92	3.85	3.09 ± 1.33	128.82 ± 55.49	8.00
	2.4^d^	2.10	2.87	3.66	2.95 ± 1.05	123.06 ± 43.95	4.00
Vitamin A (µg)							
	700^c^	336.75	385.73	450.52	397.30 ± 90.47	56.76 ± 12.92	81.60
	700^d^	329.00	382.16	436.75	387.66 ± 71.66	55.38 ± 10.24	87.00

a The nutrients are energy-adjusted using the method of residuals (44).

b RDA – recommended dietary allowance: mean daily intake level sufficient to meet the nutrient requirements of healthy individuals (45).

c Mean RDA values for women aged between 18 and 49 years (*n* = 300).

d Mean RDA values for women aged 50 years and more (*n* = 100).

e Percentage of women consuming less than 2/3 RDA values for a certain nutrient.

A 24-h recall was used to estimate the intake in terms of nutrients by using the NutriLog software.

#### Dietary iron intake and population characteristics

A multiple regression was run to predict total iron intake and iron intake adequacy from sociodemographic characteristics of the study participants (Table 4). Monthly total income was significantly positively associated with total iron intake (β = 0.18, 95% CI: 0.58, 1.97) and iron intake adequacy % (β = 0.12, 95% CI: 4.39, 16.2). Total iron intake was significantly positively associated with the education level (β = 0.14, 95% CI: 0.13, 1.74), and iron intake adequacy (%) was significantly positively associated with age (β = 0.75, 95% CI: 62.48, 76.68). The iron intake deficiency and its association with the sociodemographic characteristics are also investigated.

**Table 4:** Association of baseline sociodemographic characteristics with the normalized total iron intake and the iron intake adequacy % in the study population as assessed by multiple linear regression

Characteristics^a^	Total iron intake			Iron intake adequacy (%)		
	β	95% confidence interval (CI)		β	95% CI	
(constant)		4.84	10.87		−52.94	−0.18
Age	0.08	−0.24	1.35	0.75*	62.48	76.68
Residence area	0.03	−0.47	0.82	0.02	−4.23	6.79
Marital status	−0.02	−0.96	0.63	0.00	−6.40	6.75
University degree	0.14*	0.13	1.74	0.08	−0.41	13.31
Occupational status	−0.05	−1.19	0.52	−0.04	−10.36	4.10
Monthly total income	0.18*	0.58	1.97	0.12*	4.39	16.20

* β and 95% CI are significant at *P* < 0.05.

a All the sociodemographic variables were run in one multiple linear regression model.

Table 5 illustrates the binomial logistic regression between the iron intake deficiency (dependent variable) and the sociodemographic characteristics factors (independent variables). From the variables, only age and monthly total income added significantly to the model. The odds of having iron intake deficiency is clearly more likely among premenopausal women than postmenopausal women (OR = 171.429, 95% CI: 23.0, 1277.65). Women with lower monthly total income are approximately two times more likely (OR = 1.88, 95% CI: 1.107, 3.194) to have iron intake deficiency than women with higher income.

**Table 5:** Binomial logistic regression between iron intake deficiency and sociodemographic characteristics

Characteristics	Iron intake deficiency		
	Odd ratio	95% confidence interval (CI)	
Constant	0.007*		
**Age group^a^**			
Young adult	171.429*	23.001	1277.646
Middle-aged/old adults (rc)	1		
**Residence area**			
Rural	1.051	0.653	1.689
Urban (rc)	1		
**Marital status**			
Never married	0.795	0.449	1.409
Married (rc)	1		
**University level**			
No	1.220	0.683	2.180
Yes (rc)	1		
**Occupational status^b^**			
Housewife	0.847	0.432	1.661
Other (rc)	1		
**Monthly total income**			
≤500$	1.880*	1.107	3.194
>500$ (rc)	1		

a Young adult: aged 18–39 years old; middle aged: 39–59 years old; old adults: 60–74 years old.

b Occupational status: other means working women and students.

Rc: reference category in the binomial logistic regression.

* Significance level set at *p*-value < 0.05.

### Iron-rich dietary patterns

#### Identification of the iron-rich dietary patterns

Four iron-rich dietary patterns were obtained from the PCA, which collectively explained 38.4% of the variance in the frequency of dietary intake. Table 6 shows the factor loadings of the four patterns named according to the iron-rich food items loading highest on the respective iron-rich dietary pattern. Therefore, the patterns obtained were classified as follows: ‘legume’ pattern, which was positively associated with high-frequency consumption of beans, lentils, and chickpea; the ‘organ/Lunch meat and chicken’ pattern, as its name depicts, characterized by a high consumption of organ meat, lunch meat, and chicken; the ‘canned fish’ pattern, which was positively associated with high-frequency consumption of tuna and sardines; and the ‘beef and hummus’ pattern, as its name depicts, characterized by a high consumption of beef and hummus.

**Table 6:** Factor loading matrix for the four patterns of iron food source identified in the study population (*N* = 400), Lebanon 2018

Iron-rich food items	Iron-rich dietary pattern			
	Legumes	Organ/lunch meat and chicken	Canned fish	Beef and hummus
Beef, tenderloin, and cooked				0.712
Organ meat		0.631		
Lunch meat		0.597		
Chicken		0.591		
Tuna			0.594	
Sardines			0.765	
Egg				
Breakfast cereal fortified				
Beans	0.837			
Lentils	0.807			
Chickpea	0.665			
Hummus				0.582
Green leafy vegetables				
Dried fruit				
Roasted pumpkin seeds				
Nuts				
Carob molasses				

Rotation method: varimax with Kaiser normalization.

Kaiser–Meyer–Olkin measure of sampling adequacy = 0.656.

Bartlett’s test of sphericity <0.05 and <0.01.

Factor loadings represented are values >0.5.

#### Iron-rich dietary patterns and nutrients intake

Table 7 presents the associations of the factor scores of the various iron-rich dietary patterns with energy-adjusted nutrient intakes related to anemia. Among the four iron-rich dietary patterns, only the scores of the beef and hummus pattern had a significant and positive association with all the nutrients: protein (*r* = 0.195, *P* < 0.01), ascorbic acid (*r* = 0.193, *P* < 0.01), iron (*r* = 0.193, *P* < 0.01), folate (*r* = 0.192, *P* < 0.01), vitamin B12 (*r* = 0.194, *P* < 0.01) and vitamin A (*r* = 0.192, *P* < 0.01).

**Table 7:** Spearman’s correlation between the factor scores of the four iron-rich food patterns and the energy-adjusted nutrients intake related to iron deficiency and anemia

Nutrient^a^	Legumes	Organ/lunch meat and chicken	Canned fish	Beef and hummus
Iron	0.077	−0.084	−0.074	0.193**
Heme iron	0.075	−0.086	−0.074	0.195**
Nonheme iron	0.077	−0.084	−0.074	0.193**
Protein	0.077	−0.085	−0.074	0.195**
Ascorbic acid	0.080	−0.087	−0.074	0.193**
Folate	0.077	−0.086	−0.075	0.193**
Vitamin B12	0.075	−0.086	−0.074	0.194**
Vitamin A	0.076	−0.084	−0.074	0.192**

a The nutrients are energy-adjusted using the method of residuals (44).

** Correlation is significant at the 0.01 level (two tailed).

#### Iron-rich dietary patterns and sociodemographic characteristics

Table 8 illustrates the association between the four iron-rich dietary patterns and the sociodemographic characteristics of the study participants. Almost none of the variables was predictive for any of the iron-rich pattern except the monthly total income. A significant association was seen between the frequent consumption of canned fish and the participants with lower monthly income (β = −0.268, 95% CI: −1.215, −0.065).

**Table 8:** Association between the factor scores of the four iron-rich dietary patterns and the sociodemographic characteristics by a multiple linear regression

Characteristics^a^	Legumes			Organ/lunch meat and chicken			Canned fish			Beef and hummus		
	β	95% confidence interval (CI)		β	95% CI		β	95% CI		β	95% CI	
Constant		−1.086	0.772		−0.599	1.279		−2.476	1.943		−1.007	0.851
Age	0.067	−0.116	0.382	−0.004	−0.261	0.243	0.246	−0.136	1.208	0.098	−0.055	0.443
Residence area	0.011	−0.182	0.224	0.046	−0.112	0.298	0.077	−0.364	0.701	0.066	−0.073	0.333
Marital status	−0.028	−0.305	0.194	0.017	−0.218	0.286	−0.100	−0.900	0.457	−0.040	−0.329	0.168
University degree	0.058	−0.138	0.365	−0.103	−0.461	0.048	0.006	−0.639	0.667	−0.030	−0.312	0.191
Occupational status	0.048	−0.169	0.365	0.007	−0.255	0.284	0.111	−0.429	0.936	0.009	−0.248	0.284
Monthly total income	−0.085	−0.402	0.034	−0.063	−0.359	0.082	−0.268*	−1.215	−0.065	−0.059	−0.345	0.092

a All the sociodemographic variables were run in one multiple linear regression model.

* β and 95% CI are significant at *P* < 0.05.

### Hummus preparation and consumption

#### Common steps for hummus preparation and combination preferences with food

**Fig. 1:**
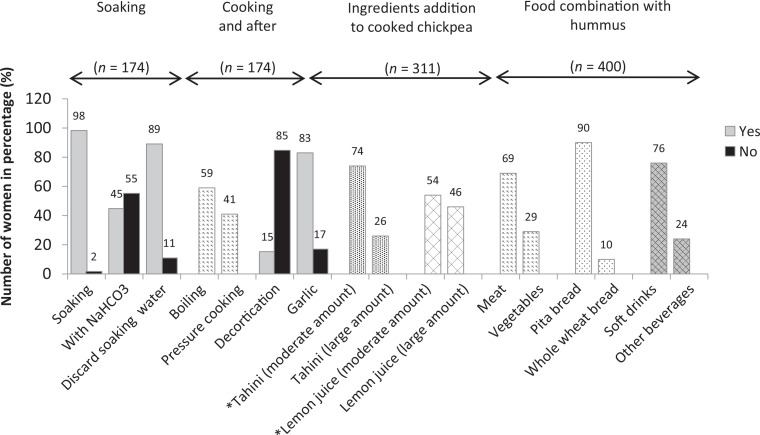
Preparation of hummus and combination with food and beverage among the studied sample (*N* = 400), Lebanon 2018. Soaking and cooking steps are reported by the women who prepare hummus traditionally (*n* = 174). Ingredient addition is reported by all the women who prepare hummus (n = 311). Food combination with hummus for consumption is reported by all the women (*N* = 400). *The amounts of tahini and lemon juice are evaluated in comparison with a traditional Lebanese recipe of hummus (47).

The preparation and the habitual food and beverage intake with hummus are reported in Fig. 1. Concerning the preparation methods, 1) soaking and discarding the soaking water are commonly practiced among the women (98 and 89%, respectively) and 2) addition of bicarbonate to soaking water is practiced by slightly less than the half (45%). The majority of the women cook using a regular pot (59%) and few of them decorticate chickpea (15%). Lemon juice and tahini are the principal ingredients added to chickpea puree. Garlic is also added by the majority (83%). Meat, an iron assimilation enhancer and a good source of iron as well (71, 72), is consumed with hummus by the majority of the women (69%). Almost all of the women (90%) prefer to consume pita bread (refined bread) with hummus more than the whole wheat bread. Pita bread may be a better option than whole wheat bread for its lower content in phytate (46). Soft drinks which are also consumed with hummus by the majority of the women (76%) are considered inhibitors of iron assimilation as food components in these beverages (*e.g.,* polyphenols and caffeine…) may decrease the absorption of iron in food (37).

#### Women who prepare hummus traditionally

The sociodemographic characteristics related to hummus preparation are shown in Table 9. More than three quarter (77.7%) of women prepare hummus, including mainly housewives and married women (93 and 88%, respectively). Ninety-one percent of middle-aged and elder women, as well as the majority of young women (67%) prepare themselves the hummus. There is no big difference between women from rural and urban areas. Income level also does not make a difference. The impact of academic level on hummus preparation is significantly important (35% do not prepare hummus, *P* = 0.004) in addition to the fact that women who work (31%, *P* = 0.001).

**Table 9:** The Lebanese women who prepare hummus traditionally or not, according to the socioeconomic and demographic characteristics (*N* = 400), Lebanon 2018

Characteristic	Prepare hummus				Do not prepare hummus		*P*
	*n* = 311 (77.7%)				*n* = 89 (22.3%)		
	Traditionally		Not traditionally				
	
	*n*	%	*n*	%	*n*	%	
**Age group^a^**							0.015*
Young adult	67	29.9	83	37.1	74	33.0	
Middle-aged/old adults	107	60.8	54	30.7	15	8.5	
**Residence area**							0.075
Rural	74	40.4	73	39.9	36	19.7	
Urban	100	46.3	64	29.2	53	24.5	
**Marital status**							0.004*
Never married	47	28.0	60	36.7	61	35.3	
Married	127	54.7	77	33.2	28	12.1	
**University level**							0.004*
No	114	60.0	60	31.6	16	8.4	
Yes	60	28.6	77	36.6	73	34.8	
**Occupational status^b^**							0.001*
Housewife	81	57.4	50	35.5	10	7.1	
Other	93	35.9	87	33.6	79	30.5	
**Monthly total income**							0.428
≤500$	71	59.1	29	24.2	20	16.7	
>500$	103	36.8	108	38.6	69	24.6	

a Young adult: aged 18–39 years old; middle aged: 39–59 years old; old adults: 60–74 years old.

b Occupational status: other means working women and students.

* Significance level set at *P* < 0.05.

#### Proper hummus preparation among women

Table 10 presents the percentage of women considering proper preparation of chickpea and hummus, and its association with sociodemographic characteristics. Less than the quarter of the women consider all the steps of proper chickpea (9.2%) and hummus preparation (22.7%). Among the variables, the age was significantly related with considering proper preparation of hummus (*P* < 0.05) with the majority being older women (66.5%).

**Table 10:** Percentage of women considering proper preparation of chickpea and hummus, and its association with sociodemographic characteristics using the χ^2^ test (*n* = 174)

	Women considering preparation with each number of proper steps									
	Chickpea,^a^ *n* (%)					Hummus,^b^ *n* (%)				
	0	1	2	3	*P*	0	1	2	3	*P*
Characteristics	4 (2.3)	88 (50.6)	66 (37.9)	16 (9.2)		16 (9.3)	54 (31.4)	63 (36.6)	39 (22.7)	
**Age group^c^**					0.36					0.00*
Young adult	3 (1.7)	36 (20.7)	23 (13.2)	5 (2.9)		11 (6.4)	26 (15.1)	15 (8.7)	13 (7.6)	
Middle-aged/old adults	1 (0.6)	52 (29.9)	43 (24.7)	11 (6.3)		5 (2.9)	28 (16.3)	48 (27.9)	26 (15.1)	
**Residence area**					0.76					0.65
Rural	2 (1.1)	37 (21.3)	30 (17.2)	5 (2.9)		6 (3.5)	21 (12.2)	31 (18.0)	16 (9.3)	
Urban	2 (1.1)	51 (29.3)	36 (20.7)	11 (6.3)		10 (5.8)	33 (19.2)	32 (18.6)	23 (13.4)	
**Marital status**					0.34					0.11
Never married	0 (0.0)	28 (16.1)	16 (9.2)	3 (1.7)		8 (4.7)	15 (8.7)	14 (8.1)	8 (4.7)	
Married	4 (2.3)	60 (34.5)	50 (28.7)	13 (7.5)		8 (4.7)	39 (22.7)	49 (28.5)	31 (18.0)	
**University level**					0.38					0.31
No	1 (0.6)	59 (33.9)	43 (24.7)	11 (6.3)		11 (6.4)	32 (18.6)	47 (27.3)	24 (14.0)	
Yes	3 (1.7)	29 (16.7)	23 (13.2)	5 (2.9)		5 (2.9)	22 (12.8)	16 (9.3)	15 (8.7)	
**Occupational status^d^**					0.49					0.59
Housewife	1 (0.6)	45 (25.9)	27 (15.5)	8 (4.6)		7 (4.1)	23 (13.4)	34 (19.8)	17 (9.9)	
Other	3 (1.7)	43 (24.7)	39 (22.4)	8 (4.6)		9 (5.2)	31 (18.0)	29 (16.9)	22 (12.8)	
**Monthly total income**					0.85					0.19
≤500$	1 (0.6)	35 (20.1)	29 (16.7)	6 (3.4)		7 (4.1)	16 (9.3)	31 (18.0)	17 (9.9)	
>500$	3 (1.7)	53 (30.5)	37 (21.3)	10 (5.7)		9 (5.2)	38 (22.1)	32 (18.6)	22 (12.8)	

a Three proper steps for chickpea preparation are soaking, pressure cooking, decortication (0: none of the steps; 1: one of the three steps; 2: two of the three steps; 3: all the three steps are considered).

b Three proper steps for hummus preparation are pressure cooking, addition of more lemon juice, and less tahini (0: none of the steps; 1: one of the three steps; 2: two of the three steps; 3: all the three steps are considered).

c Young adult: aged 18–39 years old; middle aged: 39–59 years old; old adults: 60–74 years old.

d Occupational status: other means working women and students.

* Significance level set at P < 0.05.

## Discussion

In the present survey, we aimed to assess iron intake, distinguish iron-rich dietary patterns and their association with nutrients intake and participant’s sociodemographic characteristics, and investigate whether hummus is properly prepared among 400 Lebanese women from a nationally representative sample for age and region. The study was divided into two parts.

### Iron intake data

In the first part, we assessed the nutritional intake deficiencies related to anemia and iron intake adequacy to the recommendations among the Lebanese women and its relation with the participants’ characteristics. In addition, we investigated the consumption frequency of the main dietary sources of iron and the modifiers of iron bioavailability around mealtime. Moreover, we identified the iron-rich dietary patterns and their association with nutrients intake and participant’s sociodemographic characteristics.

#### Anemia-related nutritional intake deficiencies

According to the WHO, the most important nutritional deficiencies causing anemia after iron deficiency are folate, vitamin B12, and vitamin A (48). Our study has shown that the percentage of childbearing-aged women having anemia-related nutritional intake deficiencies for iron (59%), folate (71%), and vitamin A (82%) did not improve compared with previously reported results among Lebanese women (3, 49).

#### Iron intake inadequacy among Lebanese women

Our study shows an inadequate intake of iron (65.6% of RDA) among reproductive-aged women (Table 3). This result is similar to previous national studies (3, 4, 50) and could reveal a lack of awareness, among young women, about the increased body needs for iron during the reproductive age period and the necessity to increase dietary intake of iron. Comparing with other Mediterranean countries, our result (65.6% of RDA) is similar to Jordan (51) and Spain (52), slightly higher than Egypt (57% of RDA) (53) and Greece (60% of RDA) (54), but lower than Portugal (83.3% of RDA), Italy (77.8% of RDA) (55), Croatia (88.9% of RDA) (56), and France (72% of RDA) (57). As seen, women in Lebanon like in other developing countries have lower iron adequacy to RDA values than developed countries. This could be explained by several factors such as the absence of food fortification strategies that increase iron content in staple food, the lack of awareness programs about the need to increase iron intake levels in the reproductive age period of the women, and the relatively lower socioeconomic status leading to less food diversity which contributes to better nutritional iron intake (58).

#### Iron intake and sociodemographic characteristics

The monthly income sociodemographic factor was shown to have a significant correlation with total iron intake, iron intake adequacy, and iron intake deficiency (Tables 4 and 5). This could be explained by the fact that household with lower income has been consistently associated with poorer quality of dietary intake (59). Older women were shown to have better iron adequacy and were less likely to have iron intake deficiency as the recommended dietary intake for iron for postmenopausal women is less than twice the recommended amount for premenopausal women, who have increased body needs for iron. Holding a higher university degree was positively associated with total iron intake as individuals with a higher educational level were shown in previous studies to have higher-quality diets than their counterparts (60).

#### Main sources of dietary iron

Our results show that the major sources of heme iron are beef and chicken, as reported by a previous Lebanese study (61). Nonheme iron is significantly more frequently consumed than heme iron, and its main sources reported were green leafy vegetables, legumes, and hummus. Legumes and hummus are not only good sources of iron but also complementary to meat. Our results indicate that the frequency of legumes consumption (3–6 times/week) is satisfactory to the dietary guidelines for middle eastern countries (62). Moreover, our results are similar to previously reported studies in Jordan (63) and Greece (64), where legume consumption was shown to be 3–4 times per week, and more frequent than other Mediterranean countries such as Spain and Italy (once per week) (65, 66). This signifies a preservation of legume consumption in Lebanon although the concomitant tendency to shift to more westernized diets (42). Hummus is frequently consumed (at least once/week) by the interviewed women. In the Middle Eastern countries, more than 75% of chickpea are consumed as hummus and falafel (fried chickpea patties) (67, 68).

#### Modifiers of iron bioavailability around mealtimes

According to Asmar et al. (4), consumption of tea/coffee and soft drinks among women was predictors of iron deficiency and anemia. Therefore, we have asked the women of their consumption of enhancers and inhibitors of iron bioavailability around mealtime. The frequency of consumption was higher for inhibitors around meal times than enhancers. This is supported by other studies in Lebanon (4) and other developed countries such as Brazil (69) and Australia (70). This could reveal a lacking in dietary knowledge about the consumption of iron enhancers and inhibitors around mealtimes, in both developing and developed countries.

#### Iron-rich dietary patterns

Among the four iron-rich dietary patterns obtained, legumes, organ/lunch meat chicken, canned fish, and beef and hummus, the association between beef and hummus was of interest to discuss since beef is an iron bioavailability enhancer (71, 72). The reason for their association is that hummus is eaten as a mezza and accompanies beef consumption. This was confirmed by our study as the majority of the women revealed the preference of meat consumption with hummus (Fig. 1).

#### Iron-rich dietary patterns and nutrients intake

Although hummus does not contain heme iron, its association with the beef in the same dietary pattern assigned it with a higher heme iron intake. The higher ascorbic acid associated could be due to the content of lemon juice in hummus and the accompanied consumption of ascorbic-acid-rich vegetables with hummus, as shown in our study (Fig. 1). The association with vitamin B12 and folate is due to their high content in beef (73, 74). These results are in accordance with Wallace et al. (14), showing that consumers of hummus had higher dietary intakes of vitamin A, vitamin C, folate, and iron as compared with nonconsumers (14).

#### Iron-rich dietary patterns and population characteristics

Lower household income was the only sociodemographic variable that predicted the canned fish-based dietary pattern. This is consistent with a previous study, showing that low-income households have higher consumption of canned fish compared with higher-income households that consume rather fresh food (75, 76).

### Hummus preparation and consumption

In the second part, women who prepare hummus traditionally were identified, and they were assessed for whether or not they considered preparing chickpea and hummus properly for an enhanced iron bioavailability.

#### Women who still prepare hummus traditionally

Detailed information about the women who prepare hummus traditionally is provided. Our results showing the older, married women, housewives, and women with low academic level who prepare mainly hummus are explained and comparable with other studies. According to Moisio et al. (77), food preparation is perceived as a duty and not as a self-accomplishment among senior women (77). Moreover, other studies have shown food preparation as a responsibility of the married women and housewives toward their families (78, 79). On the contrary, working women as well as women with higher academic level did not have enough time prepare to food (80, 81).

#### Proper preparation of chickpea and hummus among the women

Finally, we have investigated if the women take into consideration the steps that have been proven by literature to improve iron bioavailability from chickpea as well as hummus. As only 22.7 and 9.2% of the women reported to consider proper preparation of chickpea and hummus, respectively, this implies that the proper preparation steps of plant-based food are not well taken into account. The reason must be primarily a lack of awareness. Older women were shown to prepare hummus more properly than younger women. We believe that the reason is not related to improve iron bioavailability rather, to some culinary hacks acquired with the experience. For example, some would soak chickpea in baking soda and decorticate it as these two culinary techniques give a better texture of hummus and decrease the factors causing flatulence (82). Others would prefer pressure cooking over boiling as it reduces the cooking time of chickpea (83).

#### Hummus consumption

As for hummus combination with food and beverage, our findings report its consumption with more inhibitors (pita bread and soft drinks) than enhancers (meat), as it is the case for the higher frequency of inhibitors consumption with general food. Pita bread cannot be avoided in the Lebanese diet as it is usually consumed with all the meals (50). It is, however, better than whole wheat bread intake when iron bioavailability is in priority of consideration as it is less rich in phytate than the unrefined bread (84, 46). Therefore, hummus, like other legumes and iron-containing plant foods, must be consumed with a vitamin C-rich food source, or with at least small quantities of meat, chicken, or fish for a better iron bioavailability (37).

### Strength and limitations of the study

Our study has certain strength points as well as limitations. This is the first study that identify the iron-rich dietary patterns among the Lebanese women and that assess their consideration of the proper preparation of chickpea and hummus for a better iron bioavailability. The limitations include the execution of a single 24-h recall considering the limited time provided for the study and the lack of financial support that would allow to run blood test analysis and assess iron deficiency, iron deficiency anemia, and anemia among the women in our sample.

## Conclusion

In summary, the present study provides updated data about the nutritional intake of iron and other anemia-related nutrients among women from all Lebanese regions. It reveals that the dietary intake of iron among reproductive age women is still inadequate with about 60% of the women having iron intake deficiency with the women with lower income being the most affected. It is the first study showing the presence of four distinct iron-rich dietary patterns among the Lebanese women, with hummus-related pattern being the only one associated positively with protein, ascorbic acid, iron, folate, vitamin B12, and vitamin A intake. Moreover, it is the first to investigate whether women consider proper preparation of chickpea and hummus to improve iron bioavailability. The consumption of iron inhibitors around mealtime and hummus is frequent. Intervention programs spreading awareness among Lebanese women are needed for encouraging adequate iron intake, as well as the consumption of iron bioavailability enhancers more frequently than inhibitors around mealtimes, and finally, considering proper food preparation steps to improve iron bioavailability from plant-based food.
